# Mapping the invisible chromatin transactions of prophase chromosome remodeling

**DOI:** 10.1016/j.molcel.2021.12.039

**Published:** 2022-02-03

**Authors:** Itaru Samejima, Christos Spanos, Kumiko Samejima, Juri Rappsilber, Georg Kustatscher, William C. Earnshaw

**Affiliations:** 1 Wellcome Centre for Cell Biology, University of Edinburgh, Max Born Crescent, Edinburgh EH9 3BF, Scotland, UK; 2 Technische Universität Berlin, Chair of Bioanalytics, 10623 Berlin, Germany; 3 Institute of Quantitative Biology, Biochemistry and Biotechnology, University of Edinburgh, Max Born Crescent, Edinburgh EH9 3BF, Scotland, UK

**Keywords:** chromatin proteomics, cell cycle, prophase, mitosis, CDK1, chemical genetics, nucleolus, mitotic chromosome periphery compartment, chicken DT40 cells

## Abstract

We have used a combination of chemical genetics, chromatin proteomics, and imaging to map the earliest chromatin transactions during vertebrate cell entry into mitosis. Chicken DT40 CDK1^as^ cells undergo synchronous mitotic entry within 15 min following release from a 1NM-PP1-induced arrest in late G_2_. In addition to changes in chromatin association with nuclear pores and the nuclear envelope, earliest prophase is dominated by changes in the association of ribonucleoproteins with chromatin, particularly in the nucleolus, where pre-rRNA processing factors leave chromatin significantly before RNA polymerase I. Nuclear envelope barrier function is lost early in prophase, and cytoplasmic proteins begin to accumulate on the chromatin. As a result, outer kinetochore assembly appears complete by nuclear envelope breakdown (NEBD). Most interphase chromatin proteins remain associated with chromatin until NEBD, after which their levels drop sharply. An interactive proteomic map of chromatin transactions during mitotic entry is available as a resource at https://mitoChEP.bio.ed.ac.uk.

## Highlights

• Time-resolved dataset follows chromatin protein changes on DNA during mitotic entry
• Interphase chromatin proteins leave chromatin in sequential waves
• Dynamic changes in chromatin proteome precede prophase chromosome condensation
• Earliest changes involve ribosome biogenesis and nucleolar stress response factors

## Graphical abstract

**Figure:**
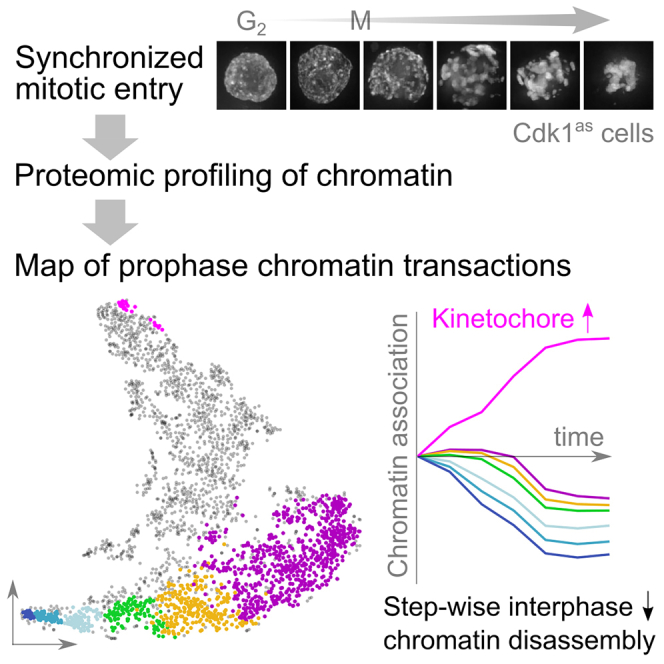

## Teaser

Using chemical genetics with analog-sensitive Cdk1, Samejima et al. map protein-DNA interactions during mitotic entry starting before rod-like chromosomes are visible. Chromatin composition evolves in orderly waves, with rRNA processing, nucleolar stress proteins, and nuclear envelope/pore proteins leaving chromatin first and lamin B1 leaving relatively late.

## Introduction

Mitotic chromosomes and interphase chromatin differ dramatically in appearance and composition. This reflects distinct functional requirements (e.g., regulated gene expression versus chromosome segregation) involving different organization of the chromatin fiber. Interphase nuclei are hierarchical ensembles of local chromatin folding (e.g., TADs) and large-scale functional segregation into compartments (Dekker and Mirny, 2016). Mitotic chromosomes are linear arrays of loops organized by nuclear condensin II and cytoplasmic condensin I (Gibcus et al., 2018; Naumova et al., 2013). Currently, little is known about how interphase chromatin structures are disassembled and how the chromatin proteome is remodeled during mitotic chromosome formation (Hirano, 2015; Paulson et al., 2021; Takahashi and Hirota, 2019; Zhou and Heald, 2020).

Mitotic entry is driven by a kinase/phosphatase network with activating and inhibitory factors shuttling between the cytoplasm and nucleus (Hagting et al., 1999). Physiologically, mitosis begins with CDK1-cyclin B1 activation on centrosomes (Jackman et al., 2003) followed by CDK1-cyclin A2-dependent migration of cyclin B1 into the nucleus (Hégarat et al., 2020). Use of a FRET reporter revealed that the earliest visible consequence of CDK1 activation in HeLa cells was cell rounding (Gavet and Pines, 2010).

Chromosome condensation is the key cytological landmark that classically defines the beginning of prophase (Flemming, 1882). Because interphase chromatin reorganization into individual mitotic chromosomes is extremely subtle at first, it is difficult to define exactly when prophase begins. Thus, the early events of mitotic chromosome formation have remained relatively inaccessible.

We have used chemical genetics to map early prophase events. Pioneering work by Shokat recognized that some kinases retain catalytic activity following replacement of a bulky “gatekeeper” residue near the ATP-binding pocket with a smaller residue (Bishop et al., 1998, 2000). This allows the inhibitor 1NM-PP1 to dock, preventing ATP binding and inactivating the kinase. 1NM-PP1 has the advantages that (1) it is highly specific for the engineered kinase and (2) it can be washed out relatively quickly and efficiently (Bishop et al., 2001; Gibcus et al., 2018; Samejima et al., 2018).

CDK1 is normally essential for mitotic entry (Nurse, 1990; Santamaría et al., 2007). Exploiting the evolutionarily conserved nature of CDK1 (Lee and Nurse, 1987), we made a sub-line of chicken DT40 cells whose cell cycle is driven by *Xenopus* CDK1^as^. *Xenopus* CDK1^F80G^ is 1NM-PP1 sensitive and retains sufficient activity to drive the growth of cultured cells (Hochegger et al., 2007). 1NM-PP1 treatment causes these DT40 cells to accumulate in late G_2_. They enter mitosis within a few minutes of inhibitor washout (Gibcus et al., 2018; Samejima et al., 2018). We previously exploited the synchronous mitotic entry of DT40 CDK1^as^ cells to study changes in the folding of the chromatin fiber during mitotic chromosome formation (Gibcus et al., 2018).

Here, we present a chromatin proteomics map of the earliest events of mitotic entry starting well before any visible sign of mitotic chromosome formation. We find that the earliest prophase chromatin changes occur at nuclear pores, on the inner surface of the nuclear envelope, and most strikingly in the nucleolus. There, proteins involved in rRNA processing move away from the chromatin, leaving behind the RNA polymerase I (RNAPI) machinery. Our work defines successive waves of chromatin proteome remodeling that accompany nuclear disassembly and mitotic chromosome formation.

## Results

### Proteomic profiling of chromatin during mitotic entry

**Figure 1:**
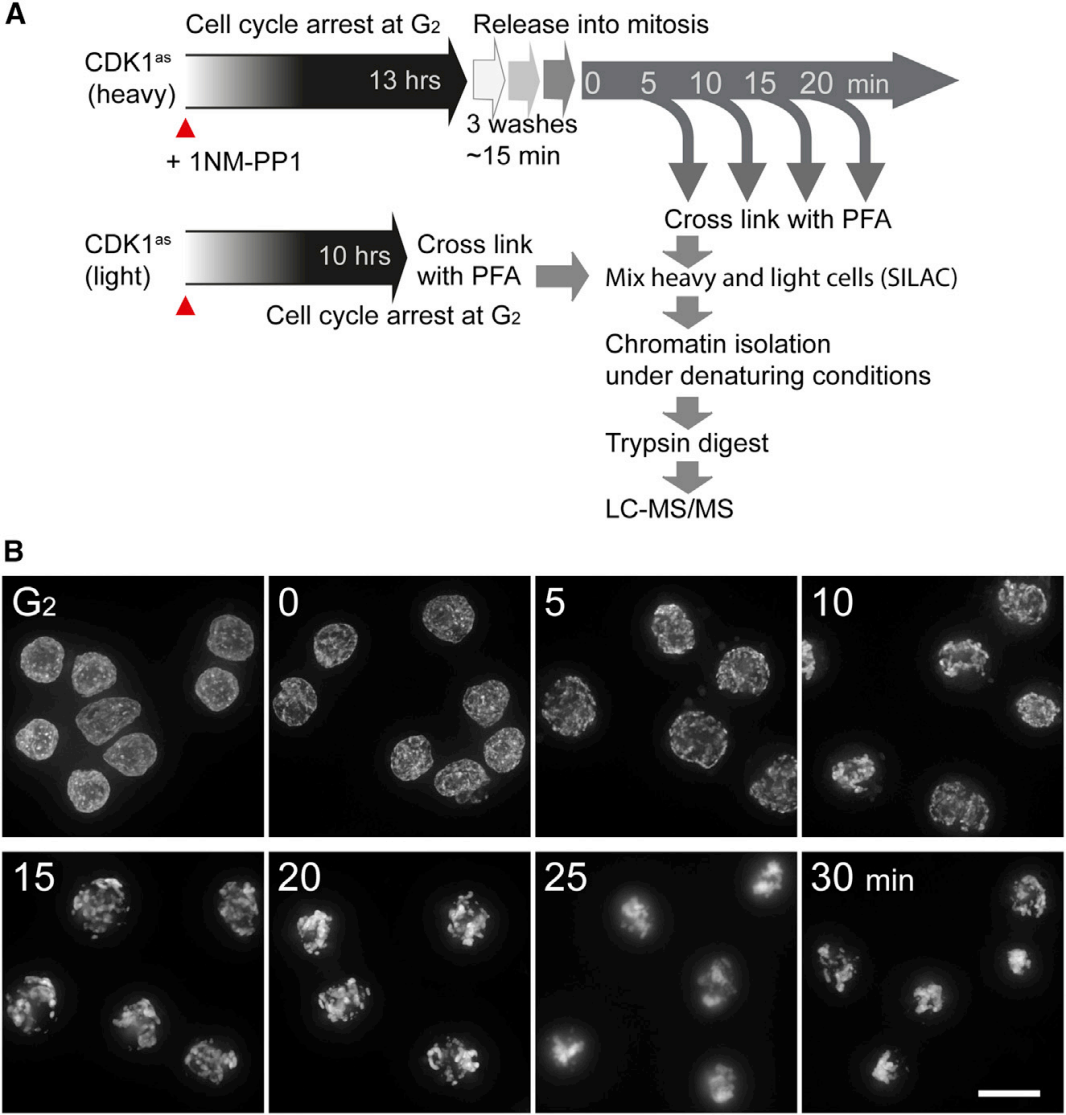
Proteomic profiling of synchronous mitotic entry (A) Workflow of time-resolved mitotic chromatin proteomics. DT40 CDK1^as^ cells arrested in G_2_ by 1NM-PP1 were released into mitosis by washout of the drug. Crosslinked cells were processed by ChEP and by LC-MS/MS (Kustatscher et al., 2014b). (B) Synchronous mitotic entry of DT40 CDK1^as^ cells. Cells were fixed with 4% formaldehyde and stained with Hoechst at the indicated times after 1NM-PP1 washout. Images are projections of z stacks. Scale bar, 10 μm.

We used a chemical-genetic system that allows us to obtain highly synchronous populations of chicken DT40 cells entering prophase (Gibcus et al., 2018) to study chromatin proteome dynamics during mitotic entry. The protocol is shown in Figure 1A.

In brief, chicken DT40 cultures whose cell cycle is driven by analog-sensitive *Xenopus* CDK1^as^ were arrested in late G_2_ with 1NM-PP1. 1NM-PP1 washout, requiring three centrifugations, activates the kinase, triggering rapid mitotic entry (Figure 1B). T = 0 in our experiments corresponds to completion of the centrifugations, ∼15 min after the first drop in 1NM-PP1 levels. We do not start our timelines with the first 1NM-PP1 washout because on different days slight differences in sample handling can cause a variation of 1–2 min in the washing time. At the end of our analysis (T = 25 min), the cells are in mid-prometaphase.

The micrographs of Figure 1B confirm the synchronous mitotic entry following CDK1 activation. The chromatin distribution is altered already by 5 min as the vast majority (> 90%) of cells enter prophase. Prophase chromosome formation is evident by 10 min. By 15 min, > 90% of the cells are in prometaphase.

These synchronous mitotic populations offer two important advantages. First, given the rapid and synchronous mitotic entry, we can study events of prophase that occur before there is any visible evidence of mitotic chromosome formation. Second, the high degree of synchrony allows biochemical analysis of events that could previously be studied only by live-cell microscopy. Our analysis uses chromatin enrichment for proteomics (ChEP), which, like chromatin immunoprecipitation (ChIP), detects the susceptibility of proteins to be formaldehyde crosslinked to DNA (Kustatscher et al., 2014a, 2014b). To measure quantitative changes with high accuracy by liquid chromatography-tandem mass spectrometry (LC-MS/MS), heavy isotope-labeled samples from each time point were combined with a light G_2_/M-arrested reference population prior to chromatin fractionation and analysis (Figure 1A) (Ong et al., 2002).

### Loss of nuclear envelope barrier function during early prophase

Changing nuclear envelope barrier function is a critical factor influencing chromatin composition during mitotic entry. Our data reveal that cytoplasmic proteins gain access to the nucleus well before visible chromosome condensation.

**Figure 2:**
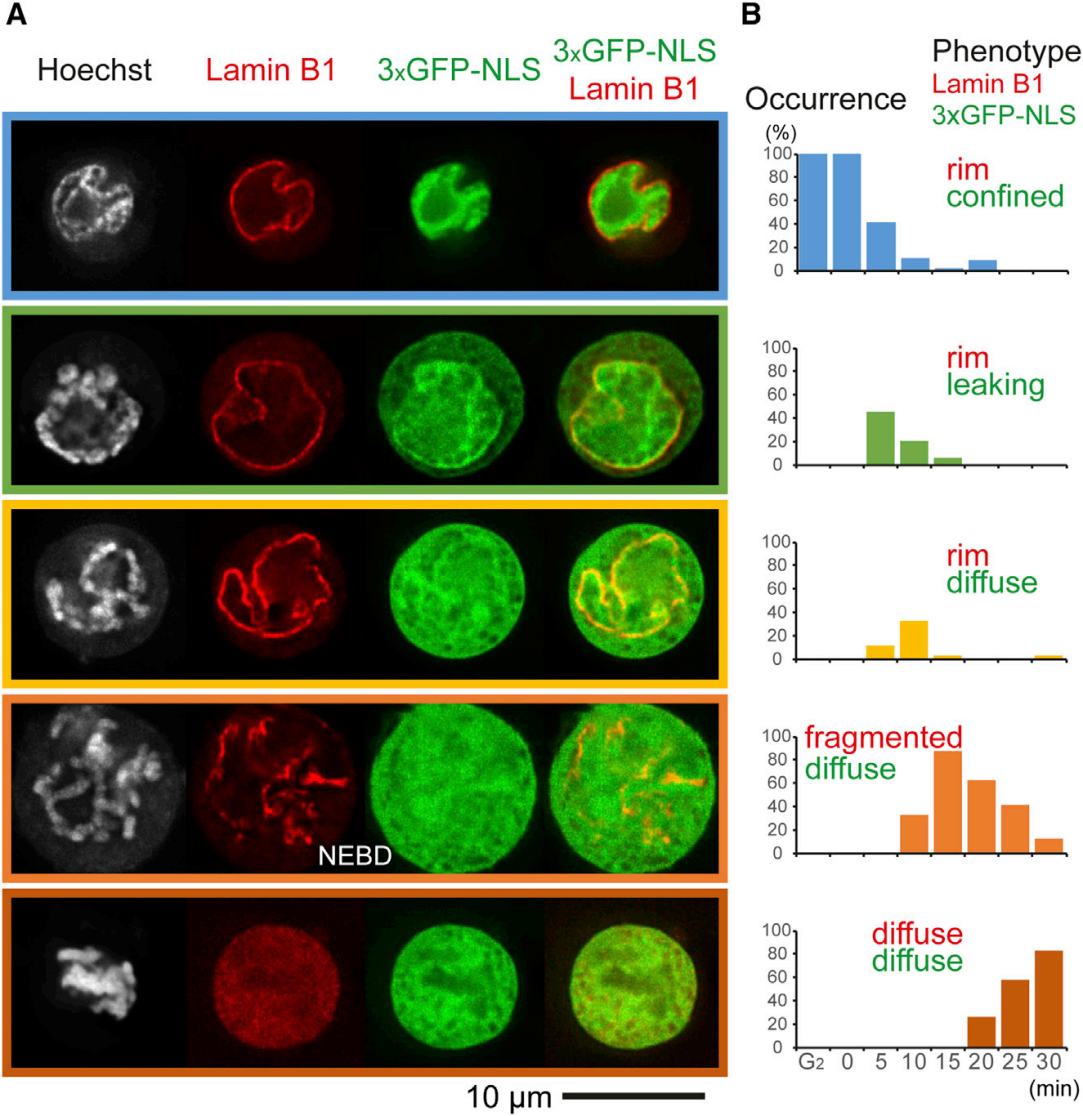
Nuclear envelope partially loses barrier function before complete breakdown (A) Cell populations at different mitotic time points were classified according to lamin B1 and GFP-NLS localization patterns. Images show single z slices of characteristic phenotypic classes. (B) Distribution of each phenotypic class along our time course.

The nuclear lamina (detected by N-terminal Halo tag knockin of lamin B1) appeared continuous at the nuclear rim in all cells from G_2_ through 5 min in our time course and in > 60% of cells at 10 min. The lamina was fragmented by 15 min in most cells, and from 20 min onward, lamin B1 was largely diffuse throughout the cell. Figure 2 shows sample images and the temporal distribution of the five phenotypic patterns observed at various time points. For simplicity we define nuclear envelope breakdown (NEBD) as the stage at which lamin B1 disassembly is observed in the microscope.

Nuclear-cytoplasmic mixing detected by leakage of 3xGFP-NLS (nuclear localization signal) into the cytoplasm occurred at 5 min in cells with an apparently intact lamina (Figures 2A and 2B, 2^nd^ row, green bars). By 10 min, the 3xGFP-NLS was evenly dispersed throughout the cell in > 60% of cells, even though lamina fragmentation was seen in only 33% of cells. Thus, the 10-min time point represents a mixture of prophase and prometaphase cells. Both lamin B1 and the 3xGFP-NLS reporter were diffuse throughout prometaphase cells (Figure 2B, bottom).

Our observation of nuclear envelope permeability prior to NEBD confirms previous results (Dultz et al., 2008; Lénárt et al., 2003) and was correlated with changes in the chromatin association of nuclear pore and inner nuclear envelope proteins (see below).

### Extensive remodeling of the chromosome proteome during prophase

**Figure 3:**
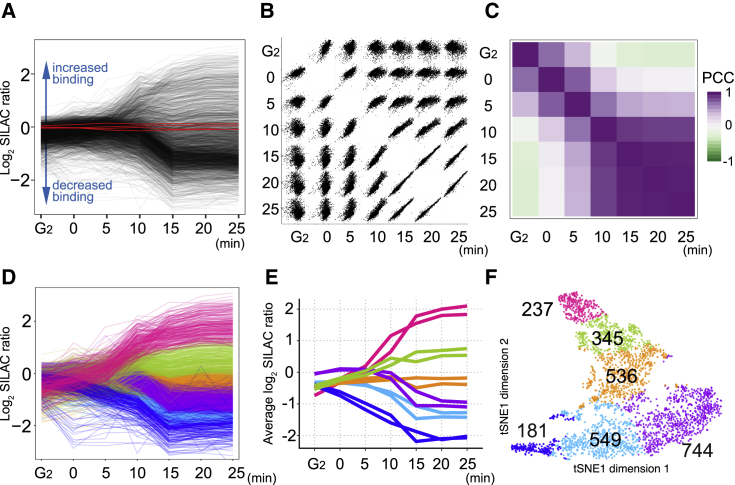
Extensive remodeling of the chromosome proteome during prophase revealed by overall patterns of chromatin protein behavior (A) General overview of chromatin protein changes during mitotic entry. Line plots showing the SILAC ratios of all proteins in the time course. Data from replicate A are shown. Red lines correspond to the core histones. (B) Scatterplots comparing SILAC ratios of all proteins between time points. (C) Heatmap illustrating Pearson correlation coefficients (PCC) between time points. (D) Line plots as in (A) color-coded according to the six *k*-means clusters. This coloring for each cluster is used in (E) and (F). (E) Line plots showing the median SILAC ratio of each cluster. Data for both replicates are shown. (F) T-distributed stochastic neighbor embedding (t-SNE) plot of the dataset in two dimensions. The numbers of proteins assigned to each of the six *k*-means clusters are shown.

**Figure 4:**
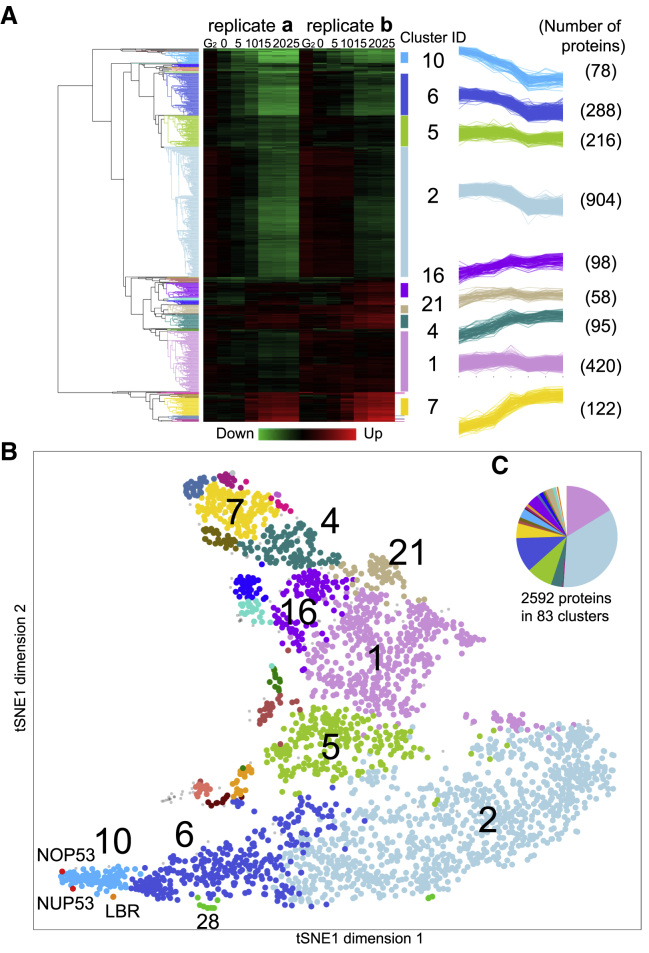
Hierarchical clustering of ChEP datasets (A) Heatmap and line plots illustrate SILAC ratio of proteins from two replicates in time course experiments. Dendrogram shows hierarchical clustering. Each cluster is highlighted using a characteristic color throughout this figure and others (Figures 5, S2, S3, and S5). Line plots show kinetic behavior of proteins in the largest nine clusters. Cluster ID and number of members are given. (B) Illustration of hierarchical clustering results on a t-SNE map. Cut tree height = 1.7. A total of 23 clusters with eight or more members and two singlets are colored, with the nine largest clusters annotated. Table S1 lists the relevant member proteins. (C) Pie chart shows the number of proteins in 83 categories by hierarchical clustering.

Our analysis identified 2,592 proteins at all time points in two biological replicates with strong reproducibility (Figure S1; Table S1). These proteins show diverse kinetic profiles (Figures 3, 4, S2, and S3). Overall, more than 1,300 (∼50%) of proteins were depleted in chromatin during mitotic entry, while more than 500 (20%) accumulated and more than 700 (29%) underwent only minor changes (numbers from clusters shown in Figure S3). Early prophase was dominated by proteins leaving chromatin, but accumulation was already evident for some by T = 5 min.

Early prophase is a time of widespread dramatic chromatin proteome remodeling that begins shortly after 1NM-PP1 washout. This is evident from the fact that protein levels of early time points (G_2_ to 5 min) are correlated poorly with each other and with all other time points (Figures 3B, 3C, and S1B). These dynamic changes cease around the time of NEBD (∼10 min, Figure 2), and proteome changes were much less widespread at the later time points (Figures 3B, 3C, and S1B). These conclusions were confirmed using principal-component analysis (PCA) (Figure S1C).

### Classifying patterns of chromosomal protein behavior during mitotic entry

To capture large trends in chromatin proteome remodeling during mitotic entry, we grouped proteins by *k*-means clustering (Figures 3D–3F). Dividing the proteomic time course into six clusters (*k* = 6) of 181–744 proteins explained 84% of the variance in the data (Figure S1D).

Most chromatin proteins significantly increase or decrease their proximity to chromatin during prophase. However, about 20% remain relatively unaffected (Figures 3D and 3E; orange cluster). This behavior was well reproduced between biological replicates (Figure 3E). The chromatin association of 181 proteins decreased strongly immediately after mitotic entry (dark blue cluster). Others decreased only after 10 min and to a lesser extent (purple cluster). As expected from Figures 3B, 3C, and S1B, remodeling of the chromatin proteome was largely complete shortly after NEBD (∼10–15 min). None of the six groups of proteins showed further significant changes after 15 min (Figures 3D, 3E, and S1B). Proteins of the purple cluster largely correspond to the “interphase chromosome proteins” identified in an earlier study (Kustatscher et al., 2014a).

We used t-distributed stochastic neighbor embedding (t-SNE) for an alternative visualization of this proteomic time course. t-SNE takes a dataset with many dimensions (e.g., two replicates, each with seven ChEP time points) and reduces it to two dimensions (Van Der Maaten and Hinton, 2008). In the t-SNE plot (Figure 3F), each point corresponds to a single protein. The proximity between points reflects how similarly the proteins behave across the mitotic progression time course. The six groups of proteins identified by *k*-means clustering occupy distinct areas of this t-SNE plot, which resembles a UK map. Proteins along the “south coast” (blue, cyan, and purple) are decreased on chromatin as cells enter mitosis, with those in the west leaving first and those in the east leaving after NEBD. Proteins in the far north (“Scotland”; magenta) accumulate on chromatin during mitosis.

To assess changes in the absolute composition of mitotic chromatin, we estimated protein copy numbers and mass using intensity-based absolute quantification (iBAQ) (Figure S2) (Arike et al., 2012; Schwanhäusser et al., 2011). In G_2_ cells, proteins of the magenta cluster account for 1.2% of the ChEP-purified material in terms of copy numbers (Figure S2A, left) and 2.1% of the protein mass (Figure S2B, left). However, these same proteins make up 8.9% (copy number) and 17.6% (mass) of the proteins associated with mitotic chromosomes, the greatest enrichment seen for any cluster (Figures S2A and S2B, right). Of the other proteins, only the green cluster was significantly increased on mitotic chromosomes. All other clusters decreased (though note that the portion of the orange cluster corresponding to core histones, marked by a black arc, remained constant).

The different prophase behavior patterns are associated with proteins having distinct biological functions. Each of the six *k*-means groups is significantly enriched for a specific set of Gene Ontology (GO) terms (Tables S1 and S2) (Ashburner et al., 2000; Gene Ontology, 2021). For example, proteins that are strongly enriched on mitotic chromosomes (magenta cluster) belong to kinetochores, the cytoskeleton, and stress granules. Conversely, ribosome biogenesis factors (blue) are rapidly depleted from chromatin during earliest prophase.

### Hierarchical clustering reveals the behavior of specific protein groups

Groups defined by *k*-means clustering are large and functionally diverse and reveal little about the behavior of specific functional groups of proteins. We therefore used hierarchical clustering for a more fine-grained analysis of the chromatin proteome.

The granularity of clustering analysis is adjusted by altering the height (h) of the cut of the dendrogram. In the analysis of Figure 4A, h = 1.7 assigned 2,592 proteins to 83 clusters: 38 clusters with 2–904 proteins and 45 with a single protein. Because we will use different values of h to reveal fine-grained features within certain clusters, we refer to cluster “X” from this level of clustering as X_/83_. Four large clusters decrease their chromatin association during mitotic entry (Figure S3A). Proteins that increase on the chromatin show a more complex behavior, with 11 distinct clusters (Figures S3B and S3C).

General trends plotted for a number of the larger clusters highlight the reproducibility of the two biological replicates (Figure S3). Two major clusters, 10_/83_ and 6_/83_, with 78 and 288 members, respectively, leave chromatin from the start of prophase (far southwest on the t-SNE map; Figure 4B). Levels of cluster 10_/83_ proteins in chromatin decline immediately upon release of cells from G_2_ arrest. The proteins of cluster 6_/83_ leave the chromatin later, after nuclear envelope permeability is compromised but with lamin B1 showing rim localization (Figures 2, 4A, and S3).

The 420 proteins of cluster 1_/83_ account for 24% of the chromatin mass at G_2_ and 27% in mitosis (Figure S2D). This cluster is dominated by the core histones, which account for 11% of the calculated protein mass in both G_2_ and mitosis. Cluster 2_/83_, with 904 members (southeast on the t-SNE map, light blue), is by far the most numerous cluster, with 35% of the proteome (Figure 4C). Its members are depleted from chromatin starting after 10 min, coincident with NEBD (Figure 2). Cluster 2_/83_ includes many “interphase chromatin proteins” (Kustatscher et al., 2014a, 2014b) and accounts for 44% of the G_2_ chromatin mass (Figure S2D). Despite the substantial decrease in their chromatin association after NEBD, these proteins remain major components of mitotic chromosomes (27% of mitotic chromatin mass; Figures S2C and S2D).

**Figure 5:**
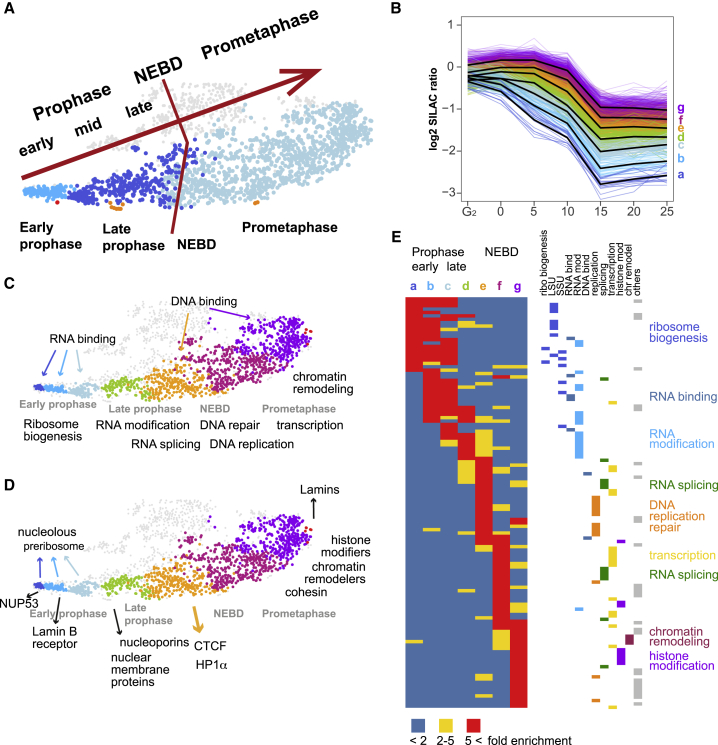
Interphase proteins leave chromatin sequentially in functional groups For a Figure360 author presentation of this figure, see https://doi.org/10.1016/j.molcel.2021.12.039. (A) Correspondence of the t-SNE map to events of mitotic progression clustered at tree height h = 1.7, as in Figure 4. Further cutting the tree height (h = 1.0) sub-divides clusters 10_/83_, 6_/83_, and 2_/83_ into seven major subclusters, shown with new coloring (a–g) in (B)–(D). (B) SILAC ratios of protein subclusters leaving chromatin. Black line: average for each subcluster. (C) GO keywords for the t-SNE distribution of (B) subclusters. (D) Examples of specific proteins and functional classes. (E) GO ribbon table (left) showing subcluster matrix at h = 1. Columns with color-coded labels show GO terms enriched in each subcluster. GO terms (rows) are color coded according to major categories. See Table S2 for details of individual GO terms.

Increasing the granularity of the analysis by setting h = 1 yielded 330 clusters (including 187 singlets). This sub-divided the 1,270 proteins of clusters 10_/83_, 6_/83_, and 2_/83_ (Figure 5A) into ten subclusters with > 10 members, plus numerous smaller clusters and singlets (Table S1 and Figure S4A). Plots using this finer-grained analysis reveal that the t-SNE map accurately reflects progressive trends early in mitosis (Figures 5C and 5D). From west to east across the south, we observed discrete waves of protein exodus from chromatin (Figure 5B). Further increasing the granularity (h = 0.6, 964 clusters/680 singlets) yielded additional information about some closely related groups of proteins (Figure S4B; see next section), but the increased number of singlets limited its utility for functional insights.

### Very few proteins are unchanged on chromatin during mitotic entry

Levels of the 420 proteins in cluster 1_/83_ change relatively little during mitotic entry. Within this cluster, 22 proteins (comprising subclusters 158_/964_ and 375_/964_) showed the least variation across the entire time course (Figure S5E). In addition to the core histones and H2A.Z, these invariant proteins comprise a very interesting group that includes kinetochore proteins (CENP-C, CENP-I, Mad1, and KNL1), CPC (chromosomal passenger complex) members (Aurora B, INCENP, borealin), shugoshin, condensin II subunits CAP-H2 and CAP-G2, SMC5, SMC6, and PP2A B56γ.

### Reorganization of interphase chromatin during mitotic entry

HMGN1 and HMGA1 are among the first proteins to leave chromatin (cluster 10_/83_). These DNA-binding proteins help regulate the higher-order structure of interphase chromatin (Catez et al., 2004; Postnikov and Bustin, 2016). However, other chromatin-organizing proteins leave the chromatin much later (cluster 2_/83_, after NEBD). These include HP1α, cohesin, HMGBs, HMG20s, chromatin modifiers, transcription factors, and mediator and integrator components (Figure 5).

In addition to regulating traffic between the nucleus and cytoplasm, nuclear pores also help regulate chromatin activity (Iglesias et al., 2020; Ptak and Wozniak, 2016; Van de Vosse et al., 2013). NUP53, a CDK1 substrate of the inner nuclear pore ring (Linder et al., 2017), is one of the earliest proteins to move away from chromatin (Figures 4B and 5D). This is followed shortly thereafter by a cluster of four nucleoporins and four nuclear inner membrane proteins. These nucleoporins, NDC1, POM121C, NUP210, NUP210L, link the nuclear pore inner ring complex to the pore membrane (Kim et al., 2018; Mitchell et al., 2010). Whether changes in inner pore ring interactions with chromatin would influence pore barrier function is not clear; however, these changes occur concomitant with weakening of nuclear envelope barrier function. Other nucleoporins that can be crosslinked to chromatin leave at diverse times after NUP53 (Figure S5B).

**Figure 6:**
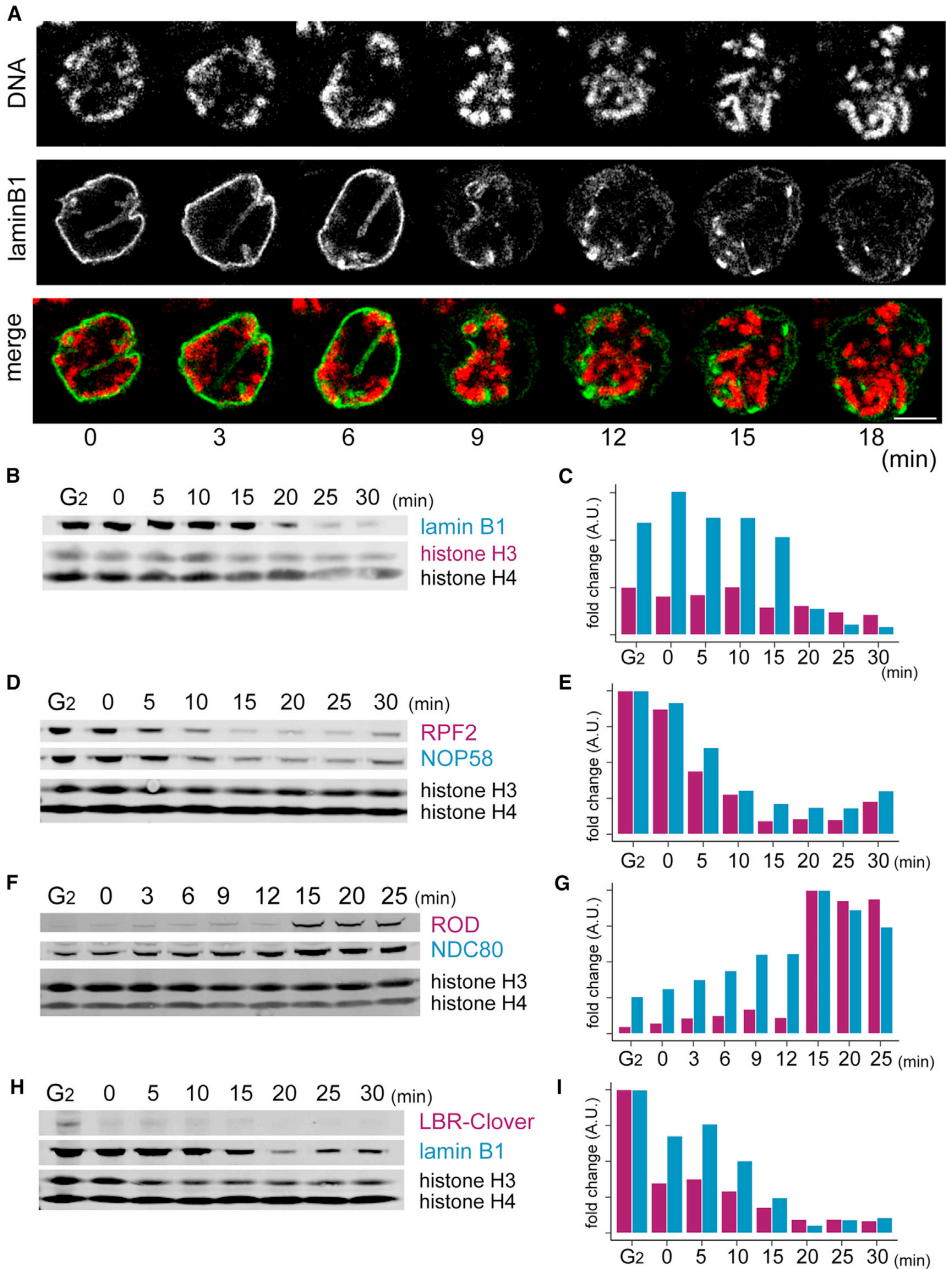
Independent confirmation of proteomics results (A) Chromosome association with lamin B1 in early mitosis. Still images from live-cell imaging of a DT40 cell expressing lamin B1 halo. DNA was stained with SiR-DNA. A single z section is shown. Bar: 5 μm. (B–I) Stepwise removal or assembly of chromosomal proteins in early mitosis. Changes reflect differential reduction or accumulation of inner nuclear membrane, nucleolar, and kinetochore proteins in ChEP chromatin. Shown are (B) lamin B1, (D) RPF2 (a LSU component), and NOP58 (an SSU component), (F) ROD and NDC80, and (H) lamin B1 and lamin B receptor (LBR). A recombinant LBR protein with Clover tag was detected by anti-GFP antibody. Histones H3 and H4 are loading controls. (C), (E), (G), and (I) are quantification of proteins shown in (B), (D), (F), and (H), respectively.

Chromatin release from the nuclear envelope is essential for mitotic chromosome formation and segregation (Champion et al., 2019). Consistent with this, the inner nuclear membrane protein lamin B receptor (LBR) also leaves mitotic chromatin very early (Figures 4B, 6H, and 6I). LBR binds both lamin B and heterochromatin protein HP1α (Ye and Worman, 1996) and may help target heterochromatin to the inner nuclear envelope. CDK1 phosphorylation was reported to antagonize LBR binding to chromatin (Courvalin et al., 1992; Takano et al., 2004). Other nuclear envelope transmembrane proteins that leave chromatin shortly after LBR include LAP2 and MAN1, which have LEM (LAP2, emerin, Man1) domains that bind the chromatin tethering/crosslinking protein BAF (barrier to autointegration factor) (Dechat et al., 2000). Thus, although HP1α- and BAF-containing heterochromatin may persist during the early stages of mitotic chromosome formation, they are apparently no longer tethered to the inner nuclear membrane.

Unexpectedly, the A- and B-type lamins comprise one of the last subclusters of interphase chromatin-associated proteins to leave the chromatin (Figures 5D and S6C). This ChEP finding was confirmed by live-cell imaging (Figure 6A) and immunoblotting of crosslinked chromatin (Figures 6B, 6C, 6H, and 6I). Although they depart somewhat later, their levels in mitotic chromatin are ultimately lower than those of most interphase chromatin proteins (Figure S6C).

### Nucleolar chromatin remodeling dominates early prophase

GO analysis reveals that proteins exit from chromatin in successive waves as cells progress through prophase. Figures 5C and 5D highlight selected individual proteins and functional classes. Surprisingly, earliest prophase is dominated by changes in the nucleolus (Figure 5E). The changes in chromatin association discussed below could reflect both disassembly of the chromatin and decreased assembly of new pre-ribosomal complexes.

The first cluster of proteins to leave chromatin (cluster 10_/83_) contains nucleolar factors involved in RNA binding and ribosome biogenesis (Figures 5B–5E, a and b [blue]). Cluster 6_/83_, which leaves slightly later, contains both nucleolar and non-nucleolar proteins (d [green], e [orange]). NOP53/PICT1 is the earliest nucleolar protein to leave chromatin, at T = 0. NOP53 is a key pre-rRNA processing factor that targets rRNA to MTR4 helicase on the exosome (Thoms et al., 2015). MTR4 and the exosome remain in chromatin until NEBD without NOP53, but pre-rRNA is presumably no longer targeted to exosomes. This likely terminates processing and assembly of the 60S large ribosomal subunit (LSU). Indeed, proteins of the LSU processome are also depleted in chromatin starting at the 0 min time point (Figures 6D and 6E). Down-regulation of pre-ribosome assembly is reinforced by early removal of c-Myc, which coordinates ribosome production via transcriptional regulation of biogenesis factors (Destefanis et al., 2020; van Riggelen et al., 2010).

**Figure 7:**
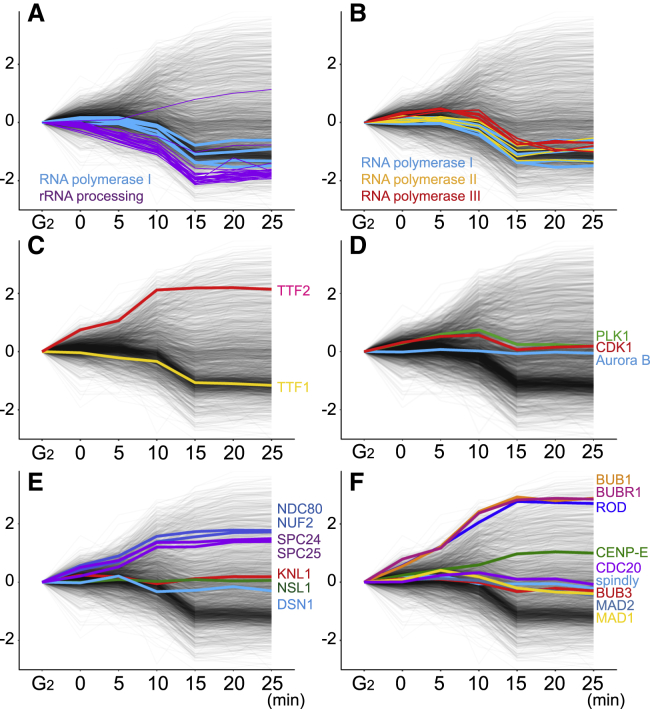
Behavior of selected proteins during mitotic entry (A–F) Profile plots of proteins of interest, including (A) ribosome biogenesis factors; (B) RNA polymerases and termination factors;(C) the mitotic kinases; (D) PLK1, CDK1 and Aurora B; and (E and F) kinetochore proteins, as indicated. Bulk proteins in the proteome are in gray.

Interestingly, RNAPI, plus several of its key transcription factors and its termination factor TTF1 (Evers et al., 1995), behave differently from the pre-rRNA processing proteins, leaving the chromatin only after NEBD (10–15 min, Figures 7A and 7C). This confirms earlier suggestions that pre-rRNA transcription continues after rRNA processing ceases, resulting in an accumulation of pre-rRNA, which ends up in the mitotic chromosome periphery compartment (MCPC) (Hernandez-Verdun, 2011; Sirri et al., 2016).

Proteins associated with mature ribosomes behave very differently compared to those involved in ribosome biogenesis (Figures S3A and S5A). They are released from chromatin early but begin to accumulate again later. This late accumulation may reflect the association of cytoplasmic hitchhikers with mitotic chromatin (cluster 17_/83_; see below).

Paradoxically, despite this mass exodus from chromatin, some proteins of cluster 10_/83_ remain among the most abundant proteins associated with mitotic chromosomes, possibly as components of the MCPC. Cluster 10_/83_ is enriched for proteins that interact with NPM1 and SURF6, two particularly abundant MCPC proteins.

### Interphase proteins leave chromatin in functional groups

Not all RNA processing factors are affected equivalently as cells enter prophase (Figure 5). As discussed above, the nucleolar chromatin and pre-rRNA processing machinery are disassembled in earliest prophase. In contrast, processes linked with RNAPII transcription are disassembled later. For example, factors involved in RNA splicing leave chromatin in late prophase, and many other chromatin factors, including histone modifiers and chromatin remodelers, leave in the general exodus that accompanies NEBD (e.g., cluster 2_/83_).

RNAPII and RNAPIII leave chromatin relatively late (Figure 7B). However, RNAPII termination factor TTF2 increases on chromatin from the start of prophase (Figure 7C), so RNAPII transcription may be shut off earlier than RNAPI transcription. RNAPII and cohesin leave the chromatin at the same time (Figure S6A), consistent with the observation that cohesin removal may regulate levels of transcription during mitosis (Perea-Resa et al., 2020). Interestingly, the three SMC protein complexes show completely distinct behaviors during mitotic entry. Cohesin leaves chromatin like a typical interphase chromatin protein, condensin I accumulates on chromatin, and the association of condensin II and SMC5/6 with chromatin does not change significantly (Figure S6B).

To detect finer patterns among the large number of proteins that leave chromatin following NEBD, we re-analyzed the data by hierarchical clustering after setting h = 0.6. This divided the dataset into 964 units: 284 subclusters and 680 singlets. The eight largest subclusters are all within cluster 2_/83_ and comprise 525 proteins (54.8% of cluster 2_/83_, 20.3% of the entire dataset) (Figure S4B, Table S1). GO terms enriched among these clusters are relevant to DNA repair, DNA replication, chromatin remodeling, histone modifications, transcription initiation, and some mRNA splicing (other splicing factors leave the chromatin earlier; Figure 5E for h = 1). The various GO subclusters tend to leave the chromatin in waves. Importantly, as stated above, although the proteins in cluster 2_/83_ reduce their abundance on chromatin, they do not vanish from it entirely. They remain as major components of mitotic chromosomes (Figure S2).

### Behavior of proteins that accumulate on mitotic chromatin is more diverse

Two protein clusters begin to associate with chromatin ahead of NEBD (Figures S3B and S3C). Both are enriched in microtubule-associated proteins, including several outer kinetochore proteins. Interestingly, the first (cluster 13_/83_) is the only cluster significantly enriched for CDK1 substrates (Figure S5C). Because most proteins in these two clusters are cytoplasmic during interphase, their accumulation on chromatin well before NEBD confirms the early loss of nuclear envelope barrier function (Figure 2).

### Kinetochore proteins show a variety of behaviors

The kinetochore is an elaborate network of multi-protein complexes that assembles at centromeres to regulate mitotic progression and chromosome segregation (Hara and Fukagawa, 2018; Navarro and Cheeseman, 2021). The CCAN and MIS12 complex (e.g., NSL1, DSN1), which comprises the centromere-proximal portion of the kinetochore, is associated with chromatin from G_2_ through to prometaphase (Figures 7E and S5E). CCAN components CENP-C and CENP-T recruit the microtubule-binding NDC80 complex (NDC80-NUF2-SPC24-SPC25) (Gascoigne et al., 2011; Nishino et al., 2013; Rago et al., 2015), which is cytoplasmic during most of interphase (Gascoigne and Cheeseman, 2013) but moves into nuclei in early prophase (Hori et al., 2003). This recruitment requires CDK1 activity (Gascoigne and Cheeseman, 2013). The NDC80 complex begins to accumulate on chromatin from time 0 in our samples (Figures 6F, 6G, and 7E). Its recruitment to chromatin is complete by 10 min (Figure 7E). Thus, kinetochores are presumably competent to capture cytoplasmic microtubules as soon as chromosomes are exposed to the cytoplasm at NEBD.

Components of the mitotic checkpoint complex (MCC) associate with chromatin in a stepwise fashion during prophase (Figure 7F). MCC components MAD1, MAD2, and CDC20 are associated with chromatin already in G_2_ cells and remain relatively invariant on chromatin throughout mitotic entry. The fourth MCC component, BubR1, is recruited later, starting in early prophase, in a cluster containing several other microtubule-associated proteins that is enriched for CDK1 substrates (Cluster 13_/83_, Figure S3B). Accumulation of MCC and other spindle assembly checkpoint components on chromatin continues even after NEBD starts.

Surprisingly, Spindly, an adaptor protein for RZZ recruitment, is stably associated with chromatin from G_2_ onward. In contrast, RZZ itself and dynactin, to which Spindly binds (Gama et al., 2017; Griffis et al., 2007), are recruited either late in prophase or after NEBD (Figures 6F, 6G, and 7F).

The astrin/kinastrin complex (Dunsch et al., 2011), which stabilizes end-on microtubule attachments at kinetochores during metaphase (Conti et al., 2019), also associates with chromatin early during prophase. Chromatin-associated astrin/kinastrin may help recruit stress granule components (Thedieck et al., 2013) to chromatin after NEBD. It may also contribute to regulating separase activity at centromeres (Thein et al., 2007). Separase also undergoes a dramatic accumulation on chromatin during mitotic entry (Figure S6A).

### Hitchhikers exhibit diverse behaviors

Hitchhikers were defined by machine learning in a previous study as proteins that are unlikely to function in chromosome formation or segregation but are physically associated with chromosomes before cell lysis and cannot be separated from them by our purification protocol (Lewis and Laemmli, 1982; Ohta et al., 2010). We believe that they usually constitute cytoplasmic proteins that stick to the highly charged chromosomes after nuclear envelope disassembly. Thus, they differ from conventional contaminants (e.g., mitochondria) that are not associated with the chromosomes *in vivo*.

Many cytoplasmic proteins accumulate on chromatin and may even plateau before lamina disassembly (Figure S3C, cluster 21_/83_). Others, e.g., cluster 7_/83_ (122 proteins), increased significantly on chromatin over our time course (from 1% in G_2_ to 11% at 20 min). GO analysis reveals that this cluster is enriched for components of clathrin- and COP-1-coated vesicles, mitochondria, cytoskeleton, and endoplasmic reticulum (Tables S1 and S2). These and several other clusters that accumulate on chromatin are significantly enriched for proteins that are more concentrated in ChEP fractions than on isolated chromosomes. Most of those proteins are cytosolic. We hypothesize that they are either hitchhikers or ChEP artifacts that are not true constituents of mitotic chromatin.

A late cluster of proteins to accumulate on chromatin is enriched in components of cytoplasmic stress granules (Figure S3B, cluster 17_/83_). Stress granules are reportedly absent from mitotic cells (Ivanov et al., 2019; Riggs et al., 2020), and polysomes disassemble during mitosis. Future studies will determine whether G3BP1, which is required to form the phase-separating scaffold that drives stress granule formation (Sanders et al., 2020; Tourrière et al., 2003; Yang et al., 2020), recruits other stress granule components to the condensing chromosomes.

## Discussion

We have mapped chromatin changes that accompany nuclear disassembly and mitotic chromosome formation using a proteomic approach based on crosslinking technology similar to that used in ChIP. We have exploited chemical genetics with analog-sensitive CDK1 to obtain near-perfect mitotic synchrony that allows us to study events in earliest prophase, before visible mitotic chromosome condensation. Our data define a sequence of chromatin remodeling events, including release of large numbers of proteins from chromatin in successive waves interleaved with the binding of cytoplasmic proteins to chromatin.

### Cytoplasmic components assemble mitotic chromatin starting early in prophase

Cytoplasmic proteins associate with chromatin prior to NEBD. Microscopy analyses confirmed that the nuclear-cytoplasmic barrier is lost within minutes of release from a G_2_ block and before visible chromosome condensation is observed. Loss of nuclear envelope barrier function during prophase was observed previously (for reference, see Dultz et al., 2008; Lénárt et al., 2003) and may be driven by CDK phosphorylation of nuclear pore components (Linder et al., 2017). Our ChEP analysis revealed that loss of barrier function correlates with movement of NUP53 and inner pore ring components away from chromatin. This process has functional consequences. For example, NDC80 complex association with chromatin during prophase can explain the extremely rapid formation of bipolar attachments by chromosomes on the spindle after NEBD.

### Surprising order of chromatin remodeling during mitotic entry

Early prophase chromatin undergoes an orderly transition as proteins leave chromatin in successive waves that form relatively tight clusters in our analysis. We expected that these early events might involve chromatin changes required to shape mitotic chromosomes. Indeed, HMGN1 and HMGA1 are two of the earliest proteins to leave chromatin. However, most other interphase chromatin components, including cohesin and components involved in chromatin modification, remodeling, transcription, and repair, only change significantly concomitant with NEBD, long after prophase chromosome formation is complete.

Chromatin release from the nuclear envelope is essential for mitotic chromosome formation and segregation (Champion et al., 2019). Indeed, early changes occurring at the nuclear periphery include chromatin release from nuclear pores and the nuclear membrane. Other nuclear envelope proteins that reduce their chromatin interactions early include LBR and several LEM-domain proteins. Nuclear pore components reportedly interact with ribosomal genes and heterochromatin (Iglesias et al., 2020; Ptak and Wozniak, 2016; Van de Vosse et al., 2013). Apparently, chromatin rich in HP1α and BAF reduces its association with the inner nuclear membrane early in prophase before loss of HP1α from chromatin (chicken BAF is not seen in our dataset). Unexpectedly, the chromatin-associated populations of lamins A and B are among the last proteins to leave chromatin in prometaphase.

The timing of protein release from chromatin is not simply proportional to the extent of direct CDK1 phosphorylation. For example, despite the presence of several known CDK1 substrates in cluster 10_/83_ (e.g., NPM1, NPM3, NCL), CDK1 substrates are not particularly enriched in this first large cluster to leave chromatin. Most CDK1 substrates change their chromatin association later in prophase and prometaphase. This appears to correlate more with the behavior of cyclins than of CDK1 itself, which shows relatively little variation in chromatin across our time course (Figure S5C). Cyclin B2 accumulates on chromatin from G_2_ onward, peaking just before NEBD. Cyclin A2 and B3 levels fall in chromatin after NEBD. CDC25A/B leave chromatin in early prophase. Cyclin B1 is yet to be identified in chicken.

### Unstressed nucleolar disassembly during mitosis

Surprisingly, GO analysis reveals that early prophase is dominated by changing associations of components involved in RNA-protein interactions including ribosome biogenesis, RNA modification, and mRNA splicing. These early changes are particularly dramatic in the nucleolus and occur long before visible changes in nucleolar structure in late prophase/prometaphase. We presume that nucleolar disassembly must occur during mitosis so that chromosomes carrying the ribosomal genes are free to segregate independently.

Inhibiting ribosome production during interphase triggers a nucleolar stress response sensed by NOP53/PICT1 (Sasaki et al., 2011) and 5S RNP (RPL5, RPL11, and 5S RNA) (Sloan et al., 2013; Weeks et al., 2019). The sensors respond by inhibiting MDM2, leading to activation of a p53-dependent pathway culminating in cell cycle arrest or apoptosis (James et al., 2014; Yang et al., 2018). A second arm of this response involves c-Myc, a master regulator of ribosome biogenesis (Destefanis et al., 2020; van Riggelen et al., 2010). A common readout of the stress response is movement of NPM1 out of the nucleolus (Yang et al., 2018).

Remarkably, one of the earliest events of prophase is movement of NPM1 away from the chromatin. However, this process does not reflect nucleolar stress as it would during interphase. Indeed, nucleolar stress sensor NOP53 is the first of the over 2,500 proteins to show a significant movement away from chromatin. Proteins found in the same cluster include c-Myc, RPF2, and RRS1. Removal of the latter two likely prevents incorporation of RPL5 and RPL11 into the LSU processome (Zhang et al., 2007). We speculate that early removal of the sensors and c-Myc from chromatin provides a mechanism permitting nucleolar disassembly without activating the stress response during mitosis.

### Complex dynamics of the MCPC

The earliest protein clusters depleted from chromatin following release from G_2_ are highly enriched in nucleolar components (clusters 10_/83_ and 6_/83_, Figure S5A). Many of these components accumulate on the surface of mitotic chromosomes in the MCPC, but this only occurs during prometaphase or even later in mitosis (Sirri et al., 2016). Indeed, the MCPC is apparently composed largely of nucleolar and pre-ribosomal proteins and RNAs (Booth et al., 2014; Hernandez-Verdun, 2011; Stenström et al., 2020). The early release of these proteins from chromatin poses an interesting conundrum. The MCPC absolutely requires Ki-67 for its formation (Booth et al., 2014; Cuylen et al., 2016; Stenström et al., 2020). However, Ki-67 leaves chromatin much later than the other nucleolar MCPC components and does not cluster with them (Figure S5A).

Many proteins of clusters 10_/83_ and 6_/83_ associate with NPM1 (Huttlin et al., 2015), which together with SURF6 drives liquid-liquid phase separation (LLPS) during nucleolar formation (Ferrolino et al., 2018). Thus, association of the nucleolar phase with chromatin is reduced long before morphological changes are evident in the nucleolus by light or electron microscopy. Many nucleolar proteins have intrinsically disordered regions that may participate in LLPS (Stenström et al., 2020), and we speculate that the MCPC represents a separated phase coating the chromosome surface. Indeed, in the absence of Ki-67, MCPC proteins form what appear to be large phase condensates in the mitotic cytoplasm (Booth et al., 2014; Hayashi et al., 2017). The location and status of these proteins between earliest prophase, when they begin to move away from chromatin together with NPM1 and SURF6, and late prophase/prometaphase, when the MCPC begins to form, remains an interesting question for future research.

### Perspectives

This map of chromatin transactions during mitotic entry has revealed several surprises. Changes in RNP associations with chromatin, particularly in the nucleolus, occur long before most changes of canonical chromatin components. Furthermore, functional remodeling of chromatin by cytoplasmic proteins occurs in early prophase long before conventional NEBD. These and other aspects of our map can be explored interactively using a dedicated app at https://mitochep.bio.ed.ac.uk.

### Limitations of the study

Abrupt full activation of CDK1 upon 1NM-PP1 washout may not perfectly mimic its natural activation in an unsynchronized cell cycle. However, CDK regulation can be modeled as a bistable switch (Kapuy et al., 2009), so this may not be a problem. Because ChEP involves formaldehyde crosslinking, it is possible that non-chromatin proteins could be captured or that some chromatin proteins could be missed. In the original ChEP study, machine learning was used to distinguish between true chromatin proteins and false “hits” (Kustatscher et al., 2014a). We include the interphase chromatin probability score from that analysis in Table S1. It is therefore unlikely that our conclusions are significantly influenced by contributions from contaminants. Our analysis only includes proteins for which identifications were obtained for all time points. Missing values were not imputed for low-abundance proteins, some of which will therefore be missing from our study.

## STAR★Methods

### Key resources table

**Table:** 

REAGENT or RESOURCE	SOURCE	IDENTIFIER
**Antibodies**		

Rabbit anti-GFP	Invitrogen	Cat# A-11122; RRID:AB_221569
Rabbit anti-lamin B1	Abcam	Cat# ab16048; RRID:AB_443298
Rabbit anti-RPF2	Atlas antibodies	Cat# HPA035475; RRID:AB_10669861
Rabbit anti-NOP58	Atlas antibodies	Cat# HPA018472: RRID:AB_1854564
Rabbit anti-KNTC1	Novus Biotechnologicals	Cat# NB100-88130; RRID:AB_1217831
Rabbit anti-NDC80	a gift from T. Fukagawa (Hori et al., 2003)	N/A
Mouse anti-histone H3	Abcam	Cat# ab10799; RRID:AB_470239
Mouse anti-histone H4	Abcam	Cat# ab31830; RRID:AB_1209246
Donkey anti-mouse IRDye 800CW	LI-COR Biosciences	Cat# 926-32212; RRID:AB_621847
Donkey anti-rabbit IRDye 800CW	LI-COR Biosciences	Cat# 926-32213; RRID:AB_621848

**Chemicals, peptides, and recombinant proteins**		

^13^C_6,_^15^N_2_-L-lysine:2 HCl	Sigma-Aldrich	Cat# 608041
^13^C_6,_^15^N_4_-L-arginine:HCl	Sigma-Aldrich	Cat# 608033
Fetal Bovine Serum	BioSera	Cat# FB1090
Dialyzed FBS (mol wt cut-off, 10,000)	Sigma-Aldrich	Cat# F0392
penicillin/streptomycin	GIBCO	Cat# 15140148
RPMI1640	GIBCO	Cat# 21875034
RPMI1640 for SILAC	Thermo Scientific	Cat# 88365
Chicken Serum	GIBCO	Cat# 16110082
1NM-PP1	a gift from J. Paulson	N/A
PIPES	Sigma-Aldrich	Cat# P1851
hygromycin B	GIBCO	Cat# 10687010
G418	GIBCO	Cat# 10131035
formaldehyde	Pierce	Cat# 28908
JF549 halo ligand	a gift from L. Davis (Chong et al., 2018)	N/A
SiR DNA	Spirochrome	Cat# SC007
Hoechst 33342	Invitrogen	Cat# H21492
Trypsin	Pierce	Cat# 90057

**Critical commercial assays**		

Quant-iT dsDNA assay kit HS	Invitrogen	Cat# Q33120
NEON transfection System 100 μl kit	Invitrogen	Cat# MPK10096

**Deposited data**		

Mass spectrometry raw data	This paper	PRIDE: PXD026385
Immunoblotting	This paper; Mendeley Data	https://data.mendeley.com/datasets/bxkkp6bv2j/3
Microscopy images	This paper; Mendeley Data	https://data.mendeley.com/datasets/bxkkp6bv2j/3

**Experimental models: Cell lines**		

Chicken DT40 cells	ATCC	CRL-2111

**Experimental models: Organisms/strains**		

DT40 cell lines with CDK1^as^ allele	Gibcus et al., 2018; Samejima et al., 2018	N/A
DT40 cell lines expressing Halo-lamin B1	This paper	N/A
DT40 cell lines expressing Halo-lamin B1and 3xGFP-NLS	This paper	N/A
DT40 cell lines expressing LBR-Clover	This paper	N/A

**Oligonucleotides**		

double strand oligos encoding BP-NLS: (*tcga*gaagcgcgtaaccgcagcggg catcacgcatccaaagaaaaag cggaaagtgtaag*ggcc* and cttacactttccgctttttctttgga tgcgtgatgcccgctgcggttacgcgcttc)	This paper	N/A
guide RNA sequence targeting Lamin B1: TCCCCTACCATCACGTCACG	This paper	N/A
guide RNA sequence targeting LBR: TGCTGAAGCACTCCATCGTT	This paper	N/A

**Recombinant DNA**		

Cas9 expressing plasmid pX330	Addgene	42230
knockin construct to insert a Halo tag at the N terminus of the lamin B1 gene	This paper	N/A
knockin construct to insert Clover in front of the stop codon in the LBR gene	This paper	N/A
Plasmid: 3Xsuperfolding GFP	Addgene	75385
Plasmid: 3Xsuperfolding GFP and NLS	This paper	N/A
Plasmid: pcDNA3	Invitrogen; Addgene	N/A

**Software and algorithms**		

MaxQuant	Cox and Mann, 2008; http://coxdocs.org/	version 1.6.7.10
Perseus	Tyanova et al., 2016; http://coxdocs.org/	version 1.6.0.7
R	https://cran.r-project.org	version 3.6.3
Image Studio	LI-COR Biosciences	version 5.2
Fiji	Schindelin et al., 2012; https://fiji.sc	version 2.1.0/1.53c
softWoRx software	Applied Precision Inc, lmage Solutions UK Ltd	version 7.0.0
Code for R Shiny app for interactive online resource	This paper	https://github.com/kustatscher-lab/mitoChEP-Shiny-App

**Other**		

An interactive online resource for analyzed data	This paper	https://mitochep.bio.ed.ac.uk

### Resource availability

#### Lead contact

Further information and requests for resources and reagents should be directed to and will be fulfilled by the lead contact, William C. Earnshaw (bill.earnshaw@ed.ac.uk)

#### Materials availability

Requests for cell lines and plasmids generated in this study should be directed to the lead contact.

### Experimental model and subject details

#### Cell lines and culture medium

The chicken lymphoma B cell line DT40 with CDK1^as^ allele (Gibcus et al., 2018; Samejima et al., 2018) was grown in RPMI1640 medium supplemented with 100 μg/mL ^13^C_6,_^15^N_2_-L-lysine:2 HCl, 30 μg/mL ^13^C_6,_^15^N_4_-L-arginine:HCl, 10% dialyzed FBS (mol wt cut-off, 10,000) and 1% penicillin/streptomycin.

GFP-NLS Halo-LaminB1 cells, LBR-Clover cells and control cells for spike-in were grown in RPMI1640 medium supplemented with complete FBS. Cells were grown at 39°C, 5% CO_2._

#### Synchronization of CDK1^as^ cells

Cells were grown to 1x10^6^ cells/ ml. 1NM-PP1 was added to 2 μM and further incubated for 10 or 13 h in medium with complete or dialyzed FBS, respectively. The G_2_ arrested cells were washed three times with RPMI medium supplemented with 56 mM PIPES pH = 7.0 (RPMI-PIPES). Washed cells were resuspended in RPMI-PIPES at cell density of 1x10^6^ cells/ ml, aliquoted and incubated for a set time.

### Method details

#### Construction of 3xGFP-NLS plasmid

DNA fragment encoding 3x superfolding GFP (digested with BamHI/XhoI) and double strand oligos encoding BP-NLS were ligated into pcDNA3 (digested with BamHI/ApaI).

#### Construction of recombinant DT40 cell lines

DT40 cell lines expressing Halo-lamin B1 or LBR-Clover were created by CRISPR/Cas9 gene editing technology. A knock-in construct was co-transfected into wild type CDK1as chicken DT40 cells using the NEON transfection system with a guide RNA and Cas9 expressing plasmid (pX330). After 24-48 h transfection, cells were transferred to 6 × 96-well plates in selective media (hygromycin 0.6-0.8 mg/ ml and/or 1.5 mg/mL G418). Expression of tags in the resultant clones were confimred by microscopy, flow cytometry and western blot analysis.

The knockin construct to insert a Halo tag at the N terminus of the lamin B1 gene contained a Hygromycin-resistant-ORF_P2A_Halo tag and 500 bp homology arms flanking the start codon. The knockin construct to insert Clover in front of the stop codon in the LBR gene consisted of 500 bp homology arms flanking the stop codon, the Clover gene and a drug (hygromycin or geneticin) resistance cassette.

In order to obtain cell lines expressing 3xGFP-NLS, Halo-lamin B1 knockin cells were transfected with plasmid encoding 3xGFP-NLS by electroporation in a GenePulser (Bio-Rad). After 24 h, cells were transferred to 4 × 96-well plates in selective media (1.5 mg/mL G418). GFP-positive clones were screened by flow cytometry and microscopy analysis.

#### Mass spectrometry

Cells were fixed with 1% formaldehyde for 10 min. To inactivate the formaldehyde, 1/20 volume of 2.5 M glycine was added and incubated for 5 min before harvesting cells. The fixed cells were washed with TBS (50 mM Tris pH 7.5, 150 mM NaCl), and snap frozen in liquid nitrogen for storage at −80°C. Once thawed on ice, heavy and light labeled cells were mixed and processed according to the ChEP protocol (Kustatscher et al., 2014a; Kustatscher et al., 2014b). In brief, formaldehyde-crosslinked cells were lysed in lysis buffer (25 mM Tris pH 7.5, 0.1% Triton X-100, 85 mM KCl). Chromatin was extracted with SDS buffer (50 mM Tris pH 7.5, 10 mM EDTA, 4% SDS), and was washed twice under denaturing conditions (6M Urea and 1% SDS), followed by a wash with SDS buffer. The DNA content of the chromatin fractions was measured using a Qubit with HS DNA QuantIT (Thermo Fisher Scientific) according to the manufacturer’s instructions.

ChEP chromatin was processed for mass spectrometry by in-gel trypsin digest. The detailed procedure is described in (Samejima and Earnshaw, 2018). The tryptic peptides were fractionated by performing strong cation exchange chromatography, using a PolySULFOETHYL A (Poly-LC) column (Hichrom, UK). Mobile phase A consisted of 5mM KH_2_PO_4_, 10% acetonitrile at pH 3; mobile phase B was 5 mM KH_2_PO_4_, 1 M KCl, and 10% acetonitrile, pH 3. The peptides were fractionated using the following gradient: 0%–60% buffer B in 18 min, then to 70% in 2 min, and then to 0% in 6 min. The flow rate was constant at 200 μl/min. Fractions were collected at 1-min time slices. Fractionated samples were combined into six fractions. The peptide samples were desalted on C18 stage tips as described before (Rappsilber et al., 2003).

Mass spectrometry analyses were performed on a Q Exactive mass spectrometer (Thermo Fisher Scientific), coupled on-line to a 50 cm Easy-Spray HPLC column ES803 (Thermo Fisher Scientific), which was assembled on an Easy-Spray source and operated constantly at 50°C. Mobile phase A consisted of 0.1% formic acid, while mobile phase B consisted of 80% acetonitrile and 0.1% formic acid. Peptides were loaded onto the column at a flow rate of 0.3 μL min^-1^ and eluted at a flow rate of 0.25 μL min^-1^ according to the following gradient: 2 to 40% buffer B in 180 min, then to 95% in 11 min (total run time of 220min).

Survey scans were performed at 70,000 resolution (scan range 350-1400 m/z) with an ion target of 1.0e6 and injection time of 20ms. MS2 was performed with an ion target of 5.0E4, injection time of 60ms and HCD fragmentation with normalized collision energy of 27 (Olsen et al., 2007). The isolation window in the quadrupole was set at 2.0 Thomson. Only ions with charge between 2 and 7 were selected for MS2.

All mass spectrometry raw files have been deposited to the ProteomeXchange Consortium (http://proteomecentral.proteomexchange.org) via the PRIDE partner repository with the dataset identifier PXD026385. The raw files were processed by MaxQuant version 1.6.7.10 (Cox and Mann, 2008) and peptide searches were conducted against the chicken reference proteome set of UniProt database (downloaded on April 2, 2020) with additional sequences from our in-house database of chicken proteins, using the Andromeda search engine (Cox et al., 2011).

#### Microscopy

Cells were fixed with 4% formaldehyde then washed with TBS (50 mM Tris pH 7.5, 150 mM NaCl) before spreading on a Polysine-coated slide. Attached cells were incubated with JF549 halo ligand (Chong et al., 2018) (a kind gift of Dr Luke Davis, Janelia Farm) followed by Hoechst 33542 (Invitrogen).

Fluorescent microscopy images were captured and processed using a legacy DeltaVison microscope system with SoftWorx software (Applied Precision Inc, lmage Solutions UK Ltd) and Fiji (Schindelin et al., 2012).

#### Live cell imaging

GFP-NLS Halo-Lamin B1 cells were treated with 1NM-PP1 for ∼13 h in normal media, then transferred to polylysine-coated glass bottom dishes (p35G-1.5-10-C, MatTek) and incubate with SiR-DNA (1/1000 Spirochrome) and Halo-JF549 (1/10,000) for ∼30 min. Just prior to image acquisition, those cells were rinsed 2 times with live cell imaging media (Leibovits L-15 media supplemented with 10% FBS and 1% Chicken serum). Images were acquired at every min using Airyscan mode on a Zeiss LSM 980 confocal, with a X 100 alpha Plan-Apochromat objective. Step size for Z stack was set to 0.3 μm. SR-4Y (max speed) and Smart set up was applied to set up the conditions. 3D datasets were visualized and analyzed using Fiji. Images show single section of 3D data stacks at every 3 min from the start of acquisition (T = 0 min).

#### Immunoblotting

DNA content in each ChEP sample was measured using a Qubit. Chromatin extracts with equal DNA amounts were loaded on NuPAGE gels (Invitrogen). The amounts of target proteins were assayed by immunoblotting followed by reading the infrared intensity of the corresponding band on the nitrocellulose membrane using an Odyssey CLx and analyzed by Image Studio ver 5.2 (Li-Cor).

### Quantification and statistical analysis

#### Data analysis

Statistical analysis was performed with R (R Core Team, 2021) and Perseus (Tyanova et al., 2016). SILAC ratios reported by MaxQuant were log2-transformed and normalized such that the average log2 ratio of the four core histones was zero at each time point. Proteins detected in half or less of the 14 analyzed samples (two replicates of seven time points) were discarded from the analysis. 2,592 of the remaining 3,500 proteins were detected in all 14 samples, and these proteins were used for statistical analyses that required complete data matrices (PCA, t-SNE and clustering).

The Rtsne package for R (Krijthe, 2015) was used to visualize the data by t-Distributed Stochastic Neighbor Embedding (t-SNE) (Van Der Maaten and Hinton, 2008). The theta parameter was set to zero to calculate the exact embedding. The perplexity parameter was set to 50, up from the default of 30, to account for the large size of the dataset.

We grouped proteins by *k*-means clustering. This divides a dataset into *k* groups based on how similar the behavior of each individual is to the mean behavior of its corresponding group across the time course. The base R function was used for *k*-means clustering, using the default algorithm by Hartigan and Wong (Hartigan and Wong, 1979).

Similarly, hierarchical clustering was performed using base R functions at standard settings (Euclidean distance and “average” agglomeration method). To call clusters at different levels of “granularity,” the clustering tree was cut at three different heights h (h = 1.7 for coarse clusters, h = 1.0 for medium clusters and h = 0.6 for fine-grained clusters).

Gene Ontology (GO) annotations for chicken were downloaded from the EBI GO Annotation Database (https://www.ebi.ac.uk/GOA/). The topGO R package (Alexa et al., 2006) was used to identify GO terms enriched in various clusters. Rather than the whole chicken proteome, only proteins that were included in the cluster analysis and had GO annotations were used as the gene ‘universe’ or background for the topGO analysis. Enrichment of GO terms in clusters was tested considering GO graph structure and using a Fisher’s exact test.

The web app that makes our results available as an interactive online resource at https://mitochep.bio.ed.ac.uk was created using R Shiny (Chang et al., 2021).

### Additional resources

An interactive proteomic map of chromatin transactions during mitotic entry is available at https://mitoChEP.bio.ed.ac.uk.

## Data Availability

• All mass spectrometry raw files have been deposited at the ProteomeXchange Consortium (http://proteomecentral.proteomexchange.org) via the PRIDE partner repository and are publicly available with the dataset identifier PXD026385. Original western blot images have been deposited at Mendeley and are publicly available as of the date of publication. The DOI is listed in the key resources table. Microscopy data reported in this paper will be shared by the lead contact upon request. • The R code required to run the app is publicly available at: https://github.com/kustatscher-lab/mitoChEP-Shiny-App. • Any additional information required to reanalyze the data reported in this paper is available from the lead contact upon request.

## Supplemental information

Document S1. Figures S1–S6

Table S1. List of proteins in ChEP samples with annotations, relevant to Figures 3, 4, 5, 6, and 7

Table S2. Results of GO enrichment analysis for each cluster and detailed list of GO terms corresponding to the rows in Figure 5E

Figure360. An author presentation of Figure 5

Document S2. Article plus supplemental information
