# Supplementary material for: Mapping the invisible chromatin transactions of prophase chromosome remodeling
Source: Mol Cell. 2022 Feb 3;82(3):696–708.e4. doi: 10.1016/j.molcel.2021.12.039 (PMC8823707; doi:10.1016/j.molcel.2021.12.039)
Supplement: Document S1. Figures S1–S6 [file mmc1.pdf]

Molecular Cell, Volume *82*

Supplemental information

Mapping the invisible chromatin transactions of prophase chromosome remodeling

Itaru Samejima, Christos Spanos, Kumiko Samejima, Juri Rappsilber, Georg Kustatscher, and William C. Earnshaw

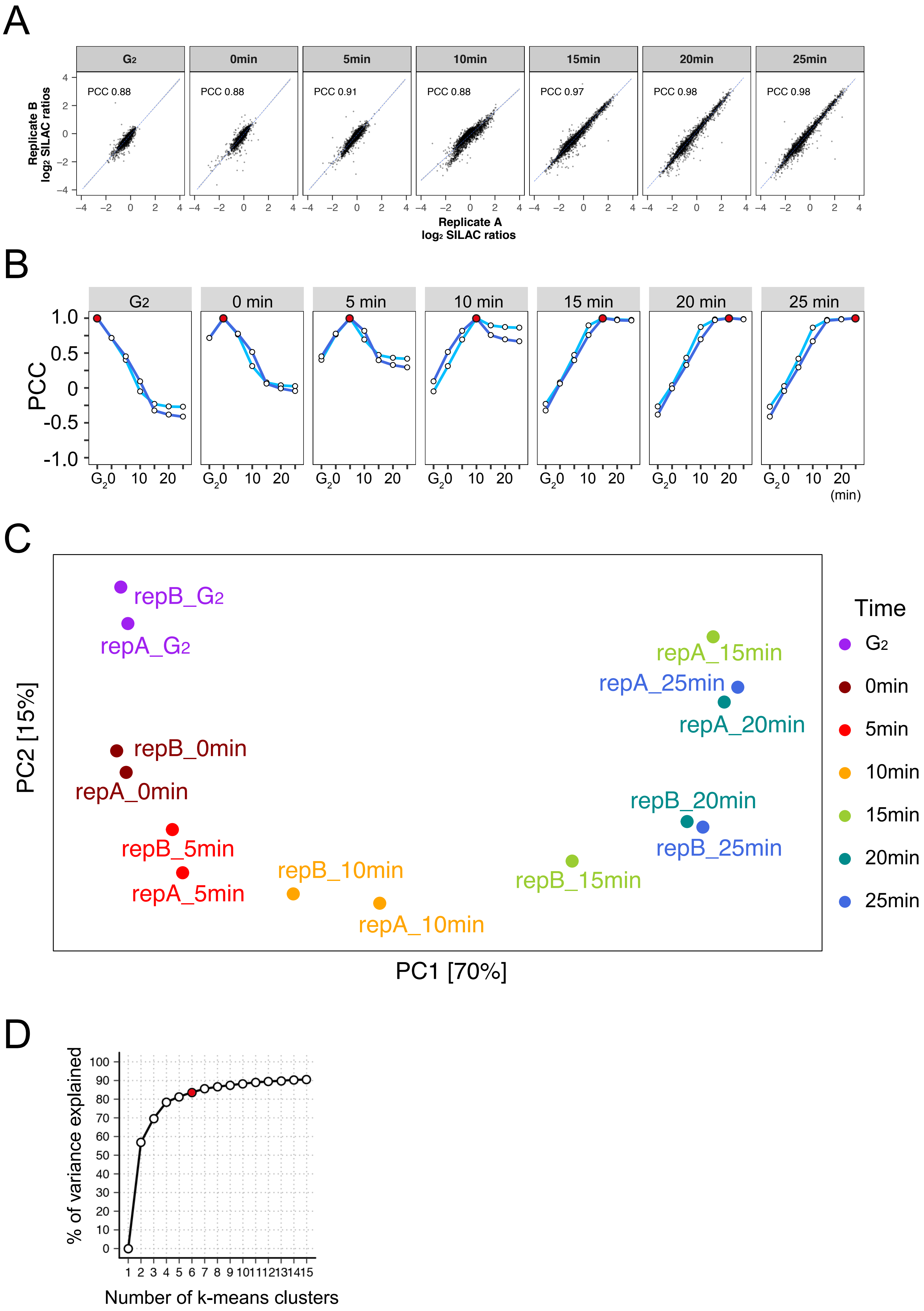
A
G2
0min
5min
10min
15min
20min
25min
PCC 0.88
PCC 0.88
PCC 0.91
PCC 0.88
PCC 0.97
PCC 0.98
PCC 0.98
Replicate B
log2 SILAC ratios
Replicate A
log2 SILAC ratios
B
G2
0 min
5 min
10 min
15 min
20 min
25 min
PCC
1.0
0.5
0
-0.5
-1.0
G2 0 10 20
(min)
C
repB_G2
repA_G2
repA_15min
repA_25min
repA_20min
repB_0min
repA_0min
repB_20min
repB_25min
repB_5min
repA_5min
repB_10min
repA_10min
repB_15min
PC2 [15%]
PC1 [70%]
Time
G2
0min
5min
10min
15min
20min
25min
D
% of variance explained
0 10 20 30 40 50 60 70 80 90 100
1 2 3 4 5 6 7 8 9 10 11 12 13 14 15
Number of k-means clusters

Figure S1. Quality control of proteomics experiment data, Related to Figure 3.

(A) Comparison of replicate A and replicate B at each time point. The Pearson Correlation Coefficient (PCC) is indicated.

(B) Comparison of proteomes from different time points. Line plots comparing the PCC between a designated time point (red dot) and all other time points. Data from two replicates are shown.

(C) Principal component analysis of the proteomic time course samples.

(D) Percentage of variance explained as a function of the number of *k*-means clusters (red circle: k=6).

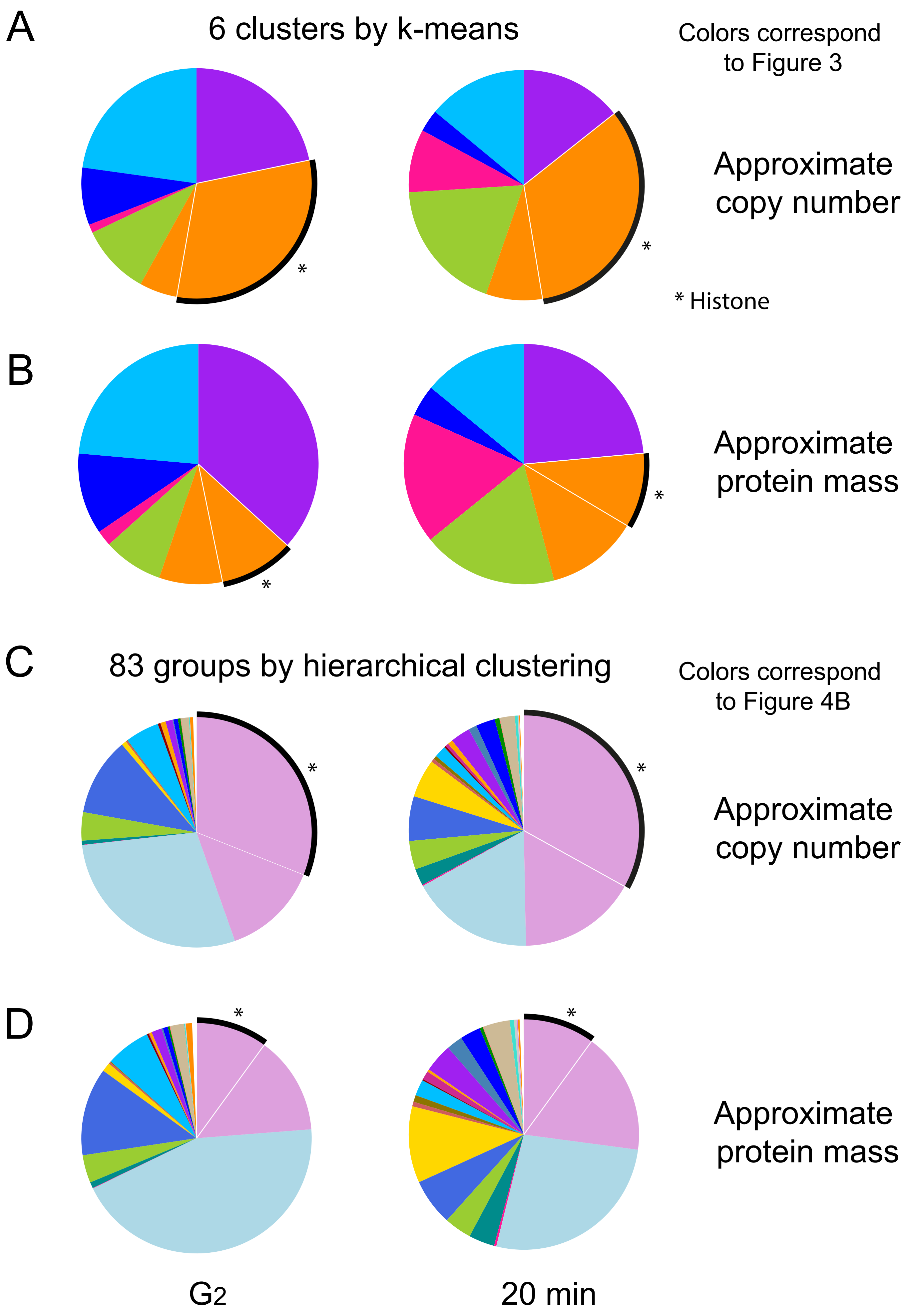

A
6 clusters by k-means
Colors correspond to Figure 3
Approximate copy number
* Histone
B
Approximate protein mass
C
83 groups by hierarchical clustering
Colors correspond to Figure 4B
Approximate copy number
D
Approximate protein mass
G2
20 min

Figure S2. Relative composition of the chromatin proteome at G_2 and in mitosis (20 min), Related to Figures 3 and 4.

(A, B) Proteins grouped by *k*-means clustering (see Figure 3)

(A) Distribution of protein copy numbers (iBAQ algorithm) in the various *k*-means clusters.

(B) Calculation of the total protein mass in the various *k*-means clusters.

(C, D) Proteins grouped by hierarchical clustering (h = 1.7 - see Figure 4)

(C) Distribution of protein copy numbers (iBAQ algorithm) in the various hierarchical clusters.

(D) Calculation of the total protein mass in the various hierarchical clusters.

The black arc with asterisks indicates the contribution of the core histones (Histone H2A, Histone H2B, Histone H3, Histone H4).

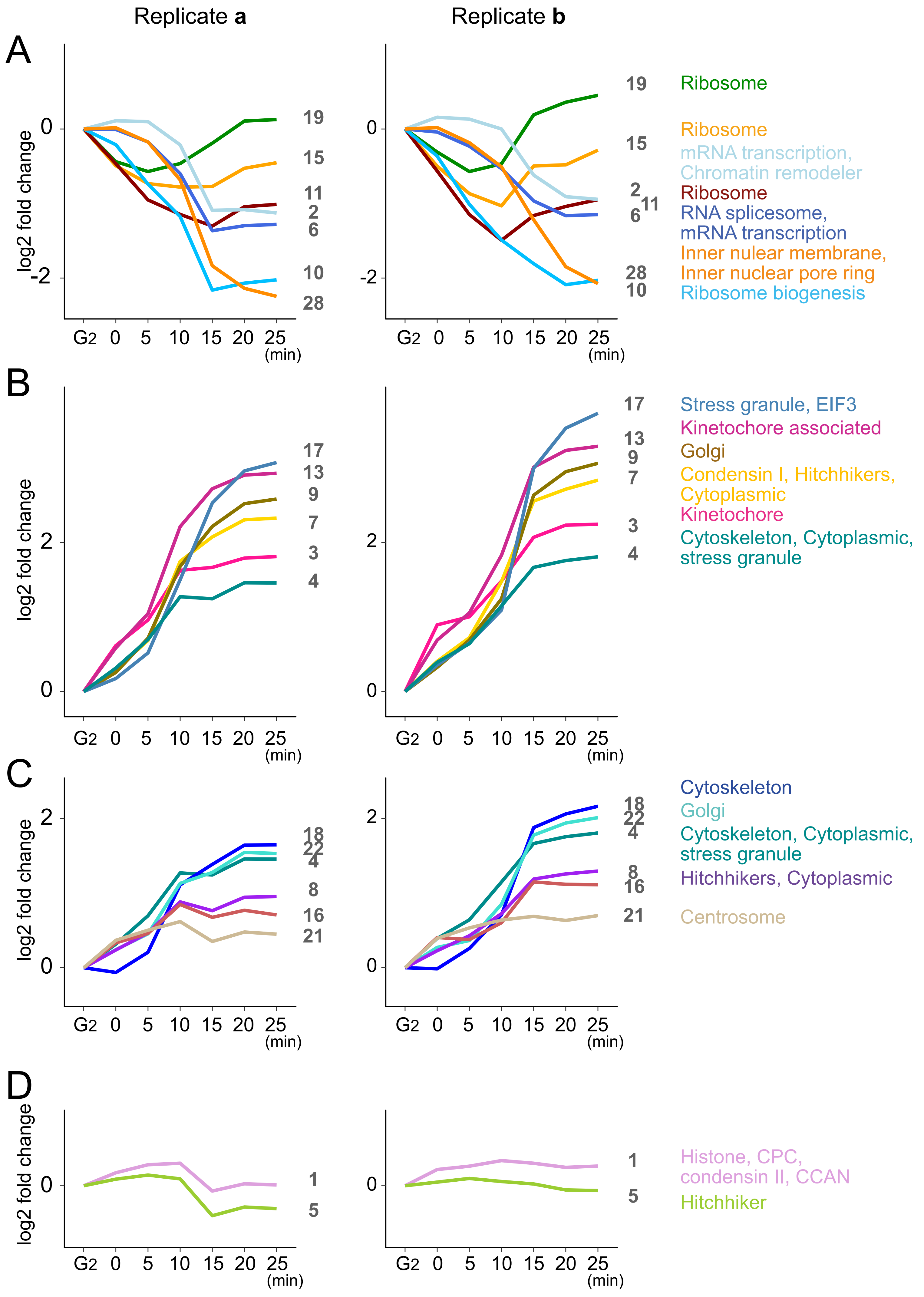

Replicate a
Replicate b
A
B
C
D
log2 fold change
G2 0 5 10 15 20 25 (min)
19
15
11
2
6
10
28
Ribosome
Ribosome
mRNA transcription, Chromatin remodeler
Ribosome
RNA splicesome, mRNA transcription
Inner nulear membrane, Inner nuclear pore ring
Ribosome biogenesis
17
13
9
7
3
4
Stress granule, EIF3
Kinetochore associated
Golgi
Condensin I, Hitchhikers, Cytoplasmic
Kinetochore
Cytoskeleton, Cytoplasmic, stress granule
18
22
4
8
16
21
Cytoskeleton
Golgi
Cytoskeleton, Cytoplasmic, stress granule
Hitchhikers, Cytoplasmic
Centrosome
1
5
Histone, CPC, condensin II, CCAN
Hitchhiker

Figure S3. Line plots of average SILAC ratio of major clusters from selected hierarchical clusters (h=1.7), Related to Figure 4.

Results from two replicates are shown in left and right columns, respectively.

A 16 clusters with >30 members at h=1

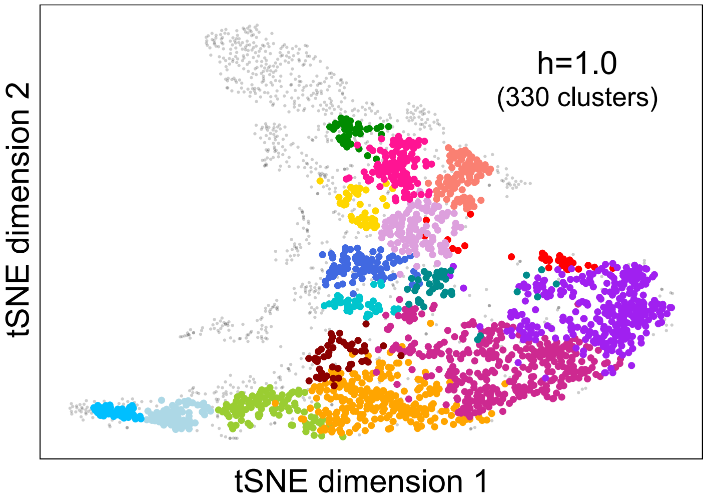

B Separation of Cluster 2/83 into 8 sub-clusters

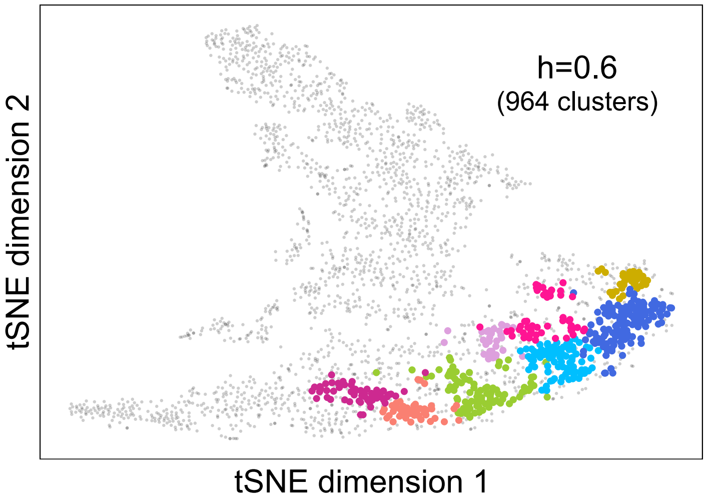

Figure S4. The largest protein clusters remaining after increasing the stringency of cut-off (decreasing the cut tree height, h) in hierarchical clustering, Related to Figure 5.

(A) At h = 1, the 16 largest $clusters_{/330}$ comprise 1661 proteins.

(B) At h = 0.6, 8 of the 9 largest $clusters_{/964}$ derive from Cluster $2_{/83}$.

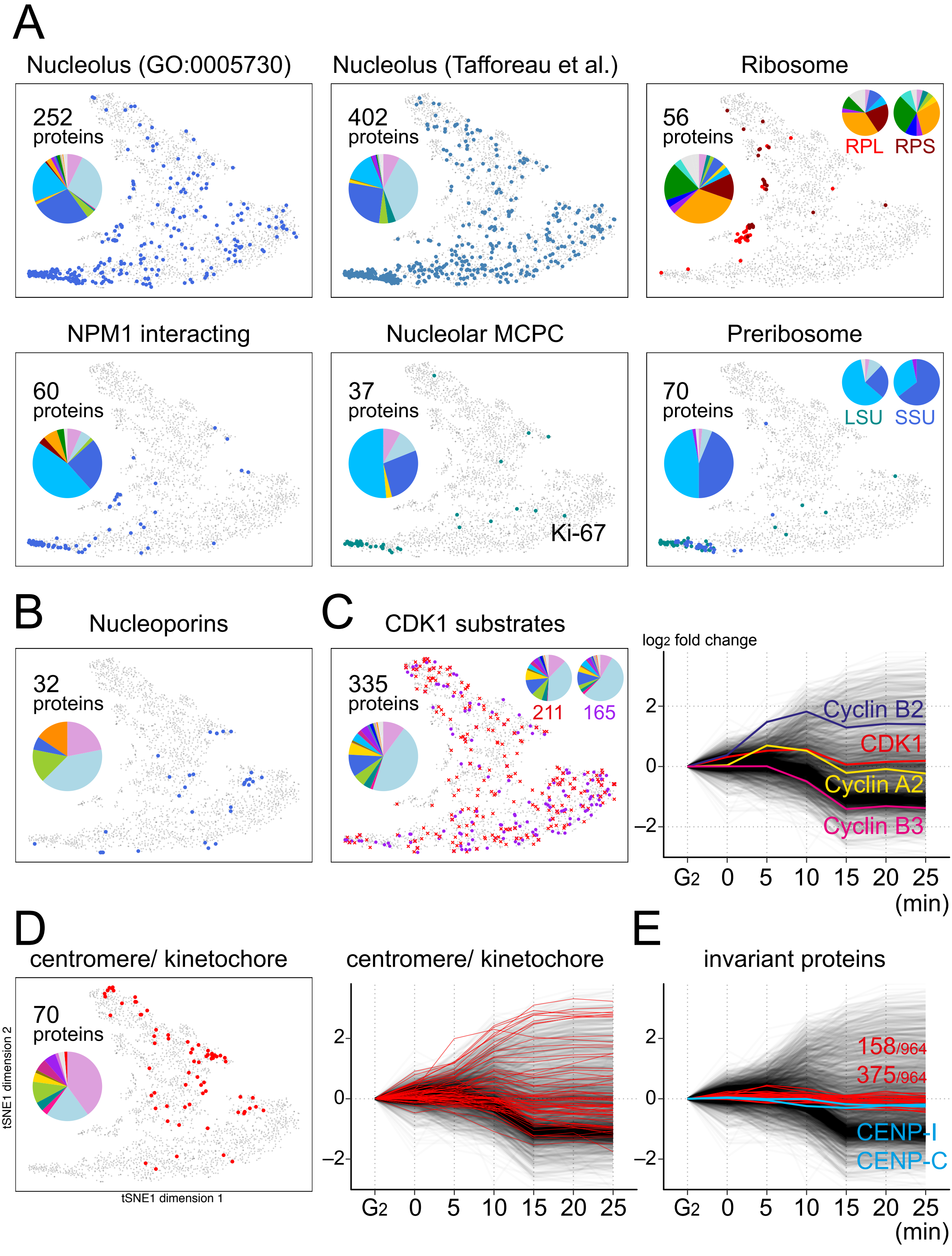
A
Nucleolus (GO:0005730)
252 proteins
Nucleolus (Tafforeau et al.)
402 proteins
Ribosome
56 proteins
RPL
RPS
NPM1 interacting
60 proteins
Nucleolar MCPC
37 proteins
Ki-67
Preribosome
70 proteins
LSU
SSU
B
Nucleoporins
32 proteins
C
CDK1 substrates
335 proteins
211
165
log2 fold change
Cyclin B2
CDK1
Cyclin A2
Cyclin B3
G2 0 5 10 15 20 25
(min)
D
centromere/ kinetochore
70 proteins
tSNE1 dimension 2
tSNE1 dimension 1
centromere/ kinetochore
E
invariant proteins
158/964
375/964
CENP-I
CENP-C
(min)

Figure S5. Behavior of selected protein groups shown by mapping on the tSNE map and hierarchical clustering, Related to Figure 5.

(A) Diversity in kinetic profiles of nucleolar proteins identified using various algorithms illustrated on the tSNE map (h = 1.7 and cluster colors as in Figure 5). The embedded pie charts show the number of proteins in each group together with their cluster affiliation at h = 1.7. See Table S1 for proteins colored-in in tSNE maps.

(B) Nucleoporins do not dissociate from chromatin in a single cluster, but are spread across the tSNE map.

(C) CDK1 substrates are spread across the tSNE map (left). Data was downloaded from PhosphoSitePlus (www.phosphosite.org) on September 21, 2021 (red crosses), and from Petrone et al. (2016) (purple circles). Only 41 proteins are common to both data-sets (lavender circles with a red cross). This suggests that the identification of CDK1 substrates in mitosis may be significantly under-saturated. (Right) Kinetic profile of CDK1 and its cyclin subunits.

(D) (left) Distribution of kinetic profiles of selected centromere / kinetochore proteins. (right) A line plot shows the changing levels of chromatin association for these proteins. Many of the proteins show relatively small changes in mitosis, but a number of them are significantly increased during mitotic entry (“Scotland” in the tSNE map).

(E) The two subclusters derived from Cluster $1_{/83}$ containing the 22 invariant proteins that include the CCAN components CENP-C and CENP-I.

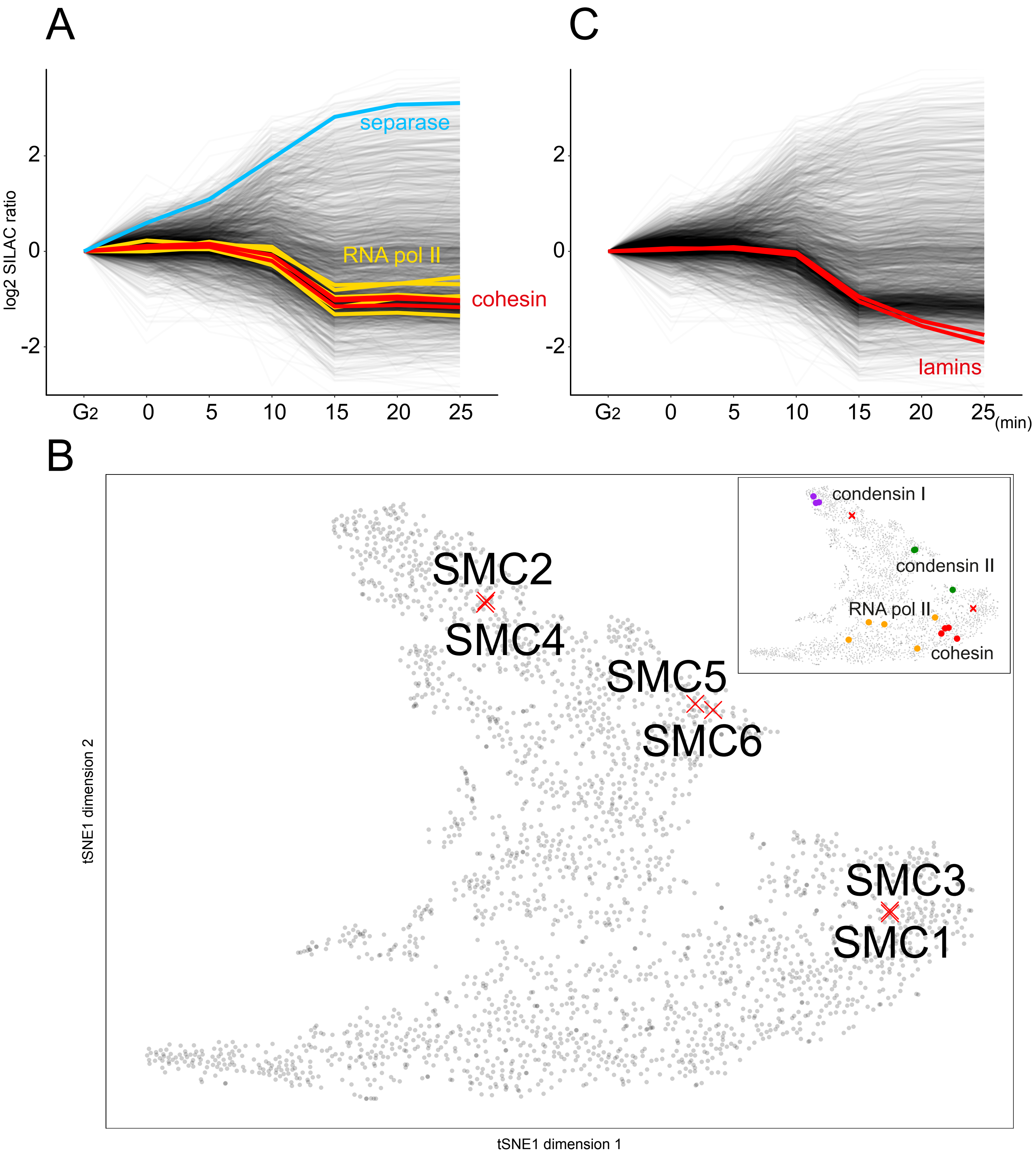

A
C
B
log2 SILAC ratio
separase
RNA pol II
cohesin
lamins
G2
0
5
10
15
20
25
(min)
2
0
-2
SMC2
SMC4
SMC5
SMC6
SMC3
SMC1
condensin I
condensin II
RNA pol II
cohesin
tSNE1 dimension 2
tSNE1 dimension 1

Figure S6. Behavior of selected proteins during mitotic entry, Related to Figure 7.

(A) Line plots of all proteins in the time course ChEP proteome. Highlighted are separase (light blue), subunits of cohesin (red) and subunits of RNA polymerase II (yellow).

(B) Positions of SMC proteins in tSNE map. (inset) non-SMC subunits of condensin I (purple), condensin II (green) and cohesin (red). RNA polymerase II subunits are shown in orange circles. Red crosses are SMC1 - SMC4 as shown in the main map.

(C) Line graphs of every protein in the time course ChEP proteome. Lamins A and B1 are highlighted in red.
